# Intestinal obstructional outcome in paediatric age group: A general surgeon's prospective

**DOI:** 10.6026/973206300223732

**Published:** 2026-06-30

**Authors:** Ritesh Yadav, Akhilesh Ratnakar, Sourabh Jain, Talha Saad, Harsh Kumar Goyal, Amar Gangwani, Shashi Marko

**Affiliations:** 1 Department of General Medicine, Shrimant Rajmata Vijayaraje Scindia Medical College & Hospital, Shivpuri, Madhya Pradesh, India; 2 Department of General Surgery, Bundelkhand Government Medical College, Sagar, Madhya Pradesh, India; 3 Department of Emergency Medicine, Bundelkhand Government Medical College, Saga, Madhya Pradesh, India; 4 Department of Respiratory Medicine, Bundelkhand Medical College Sagar, Madhya Pradesh, India; 5 Department of Surgery, Bundelkhand Medical College, Sagar, Madhya Pradesh, India; 6 Department of Surgery, Bundelkhand Government Medical College, Sagar, Madhya Pradesh, India; 7 Department of Pharmacology, Bundelkhand Government Medical College, Sagar, Madhya Pradesh, India

**Keywords:** Intestinal obstruction, paediatric patients, congenital type of lesion

## Abstract

Intestinal obstruction is one of the most common surgical emergencies seen in children. Therefore, it of interest to evaluate the pattern of disease occurrence, clinical presentation and treatment outcomes in children with intestinal obstruction. Infants (31.3%) are highly affected by intestinal obstruction than other age groups. Boy child exhibits number cases of intestinal obstruction than girl child. Imperforated anus (52.2%) is the most common congenital types of lesions causing the disease; Intussusception (69.5%) is the commoner acquired lesion and abdominal distention (32.8%) is the typical feature which was exhibited by many child cases. Thus, data shows the best outcome, early diagnosis and intervention are key essentials and hence will facilitate in enhancing knowledge on reduction of mortality rate of the cases.

## Background:

Intestinal obstruction is one of the most frequent surgical emergencies affecting children. In recent years, the approach to diagnosing and managing this condition has improved considerably. These advances are the result of a deeper understanding of the disease process, progress in diagnostic technology, the availability of more effective antibiotics, safer anaesthetic practices for children and better intensive care facilities before and after surgery [1]. Although the basic mechanism of intestinal obstruction in children is similar to that in adult, congenital abnormalities contribute far more significantly in the paediatric age group. Children are also particularly sensitive to disturbances in fluid and electrolyte balance, making careful monitoring and correction an essential part of early management. The causes of intestinal obstruction in children are varied and show marked differences across regions and countries [2]. Several conditions are commonly responsible for obstruction in paediatric patients, including intussusception, infantile hypertrophic pyloric stenosis, Hirschsprung's disease, imperforate anus and meconium ileus, malrotation with volvulus, intestinal atresia and annular pancreas [3]. Most children present with symptoms such as abdominal swelling, vomiting, constipation or failure to pass meconium and signs of dehydration or electrolyte imbalance [4]. Many congenital causes become evident during the neonatal period, while infantile hypertrophic pyloric stenosis usually appears around the third week of life. Intussusception is most often seen in healthy infants between six and eleven months of age. The timing and pattern of symptom onset can also help in narrowing the diagnosis. For example, intussusception and volvulus typically present suddenly, whereas Hirschsprung's disease often shows a more gradual and intermittent course [5]. Despite the availability of advanced facilities in developed settings and select centres locally, a large proportion of children requiring surgical care are still managed by general surgeons. This emphasises the importance of being familiar with the wide range of clinical presentations and the unique physiological responses of children to illness and surgical intervention. Early recognition and timely treatment are vital, as delays can lead to serious complications such as severe fluid and electrolyte imbalance, bowel perforation, peritonitis, aspiration pneumonitis and even death [6]. Therefore, it is of interest to describe the clinical and pathological features of the various causes of intestinal obstruction in children, to determine the most common conditions across different age groups and to anticipate perioperative challenges and complications.

## Materials and Methods:

## Research design and sample:

The observational research was conducted prospectively over 2 years. This research was executed on 67 patients, who were computed utilising the following formula. Child patients aged between 1 day and 12 years who were suffering from intestinal obstruction and were hospitalised in the department of surgery for the condition were included in the present research. Child patients whose definitive diagnosis of intestinal obstruction was unclear during investigations were excluded from the study. Child subjects who underwent LAMA or escaped before we could determine the cause of intestinal obstruction were also excluded from the current research. Every safety measure was implemented to ensure that research participants' personal information remained private and protected. The ideals of the Declaration of Helsinki guided our research. Every subject gave their informed consent. The study received ethical clearance from the relevant ethical committee. To proceed, we estimated the subjects' size using the aforementioned formula. Equation is N = z^2^ x p^ (1 - p^) / ^2^, wherein z refers to the z score, N refers to the size of the population and p̂ refers to the population proportion. The population proportion was assumed to be 50%, the margin of error was presumed to be 12% at a 95% confidence interval and the sample size came out to be 67. We conducted our research on 67 child patients. For subjects who came to the department with symptoms of intestinal obstruction and presented the signs of the same, an intestinal obstruction diagnosis was established by abdominal radiograph or an invertogram. All subjects underwent a variety of imaging techniques, including computed tomography, magnetic resonance imaging, barium study and ultrasonography. A proforma was outlined for the collection of clinical and epidemiological data. In accordance with standard protocol, child subjects were treated with the utmost care. All blood investigations were analysed, including a complete blood count, serum electrolytes and urea levels. All the child patients were saved initially until their conditions improved. The child patients were then arranged into those who require an operation and those who can be managed without an operation. All child subjects were observed with regard to their age, gender, causes of intestinal obstruction, including the types of lesions, clinical features of the intestinal obstruction and post-procedural challenges.

## Results:

Amongst the lesions, congenital causes accounted to 44 and acquired causes in 23 of them. In the congenital causes, majority of the cases were imperforated anus seen in 52.2% of the patients. This was followed by Hirschprung's disease (20.4%), Meckel's diverticulum (11.3%), Jejunal / ileal atresia (6.8%), hypertrophic pyloric stenosis in 2.2%, malrotation in 4.5% and giant cystic meconium peritonitis in 2.2%. In the acquired causes, intussusception accounted to 69.5% of the causes as seen in Table 1. Abdominal distension was the most commonly seen clinical features with 22 of them reported with it while abdominal pain and vomiting and abdominal pain were seen in least cases. The most commonly noted post procedural complication was excoriation of skin seen in 41.6% of patients.

## Discussion:

Intestinal obstruction is one of the most commonly seen surgical emergencies in children and forms a large part of paediatric surgical admissions. Unlike adults, in whom post-operative adhesions and tumours are the usual causes, children most often develop intestinal obstruction due to conditions such as intussusception and obstructed external hernias. Depending on the age at which it occurs, paediatric intestinal obstruction is broadly classified into neonatal and non-neonatal types. The causes of this condition vary widely across different regions, with noticeable differences reported between countries and geographical areas. In the present research, intestinal obstruction was greatest in infants (1 month - 1 year of age) which were followed by neonates. Batra *et al.* described similar results in their research; they also discovered that infants were highly affected by intestinal obstruction [7]. The findings of the research are inconsistent with the investigation done by Singh *et al.* who reported that neonates were highly affected [4]. In the current research, males (55.2%) were highly affected by intestinal obstruction in juxtapose to females (44.7%). These findings are consistent with the outcomes observed by other studies [4, 7]. Memon *et al.* and Liu *et al.* also reported the similar findings with male to female ratio 2:1 and 4.4:1 respectively [8, 9]. The similar type of research was reported by Rani *et al.* and Talpade *et al.* as well [10, 11]. In this current investigation, clinical features of the intestinal obstruction were assessed which resulted in abdominal distension (22 child cases) followed by meconium (19 child cases), excessive crying (17 child cases), visible peristalsis (5 cases), abdominal pain (2 cases) and vomiting (2 cases). Similar studies also reported that vomiting was the typical symptom of the child cases in his research followed by constipation and abdominal distension [12, 13]. In the present investigation, 17.9% of the child cases exhibited the complications after surgery. Out of 17.9% of the cases, excoriation of the skin was the highest which was subsequently followed by wound infection and septicaemia. Only 1 child case exhibit retraction of colostomy. Literature evidence also presents similar findings [10, 11, 14]. The mortality rate in the present research was 10.4% (7 child cases). Toddler age group had the highest mortality rate in this study. The most typical reasons of the intestinal obstruction were either congenital or acquired. In the present research, type of acquired lesion affected the 23 child cases. Out of these 23 child subjects, 69.5% of the child patients had intussusception, 27.5% of the cases had abdominal tuberculosis and 4.3% child cases had worm obstruction and gangrenous appendicitis consecutively. It was uncovered that intussusception is the most typical acquired cause of the intestinal obstruction in child patients. These findings were consistent with another study [6]. In this current investigation, congenital causes of intestinal obstruction were 65.6%. Out of all the congenital causes, imperforated anuses were the commoner (23 child cases). 9 cases were suffering from Hirschprung's diseases type of lesion. 5 cases had the congenital lesion known as Meckel's diverticulum. This implies that imperforated anus were the common congenital type of lesion discovered in our research [15].

## Conclusion: 

Infants were the most often affected age group, with a greater prevalence found in males. Imperforate anus and intussusception were the primary congenital and acquired causes of intestinal blockage, respectively, with abdominal distension being the most common presenting symptom. Early identification and timely therapy by trained clinicians are critical for improving outcomes and lowering mortality.

## Figures and Tables

**Table 1:** Distribution of study population based on type of the lesion, clinical features and post-procedural complications

** Type of lesion (N = 67)**		**Number**	**Percentage**
Congenital causes	Imperforated anus	23	52.20%
(n = 44)	Hirschprung's diseases	9	20.40%
	Meckel's diverticulum	5	11.30%
	Jejunal/ileal atresia	3	6.80%
	Hypertrophic pyloric stenosis	1	2.20%
	Malrotation	2	4.50%
	Giant cystic meconium peritonitis	1	2.20%
Acquired causes	Intussusception	16	69.50%
(n = 23)	Abdominal tuberculosis	5	21.70%
	Gangrenous appendicitis	1	4.30%
	Worm obstruction	1	4.30%
Clinical Features (N = 67)		Number	Percentage
	Abdominal distension	22	32.80%
	Not passing stool/ muconium	19	28.30%
	Excessive crying	17	25.30%
	Abdominal pain	2	2.90%
	Visible peristalsis	5	7.40%
	Vomiting	2	2.90%
Post- procedural complications (n = 12)		Number	Percentage
	Wound infection	3	25%
	Excoriation of skin	5	41.60%
	Septicaemia	3	25%
	Retraction of colostomy	1	8.30%
